# Fitness, motor competence and body composition as correlates of adolescent neck/shoulder pain: an exploratory cross-sectional study

**DOI:** 10.1186/1471-2458-8-290

**Published:** 2008-08-15

**Authors:** Mark C Perry, Leon M Straker, Peter B O'Sullivan, Anne J Smith, Beth Hands

**Affiliations:** 1 School of Physiotherapy, Curtin University of Technology, Perth, Australia; 2 Centre for Sports and Exercise Medicine, Barts and the London School of Medicine and Dentistry, London, UK; 3 School of Health Sciences, Notre Dame University, Perth, Australia

## Abstract

**Background:**

Adolescent neck/shoulder pain (NSP) is a common and sometimes debilitating problem. Several risk factors for this condition have been investigated, but no studies have previously evaluated associations between fitness, motor competence, body composition and adolescent NSP.

**Methods:**

1608 males and females of mean age 14 years answered questions on their history of NSP (4 measures), and were tested for aerobic fitness, upper and lower limb power, trunk endurance, grip strength, shoulder flexibility, motor competence and anthropometric factors. Univariate and multivariate logistic regressions were used to test for associations between NSP and physical variables.

**Results:**

There were significant gender differences for most physical and pain variables. After multivariate analysis, males had lower odds of NSP if they had reduced back endurance [OR: 0.66 (95% CI: 0.46–0.97)], reduced persistent control [0.42 (0.19–0.95], and increased muscle power [0.33 (0.12–0.94)], and higher odds of NSP if they had a higher basketball throw [2.47 (1.22–5.00)] and jump performance [3.47 (1.55–7.74)]. Females had lower odds for NSP if they had a reduced jump performance [0.61(0.41–0.92)], a better basketball throw [0.60(0.40–0.90)], lower shoulder flexibility [0.54 (0.30–0.98)] and a higher aerobic capacity [0.61 (0.40–0.93)], and higher odds for NSP if they had greater abdominal endurance [1.57(1.07–2.31)] and greater bimanual dexterity [1.77(1.18–2.65)]. Females showed a U shaped relationship between NSP and back endurance [low: 2.12 (1.20–3.74); high 2.12 (1.18–3.83)].

**Conclusion:**

Adolescent NSP was associated with fitness and motor competence, although the associations varied with gender, and their strength was limited.

## Background

Neck/shoulder pain (NSP) may affect up to half of adolescents [1], leading to significant loss of function [2]. Up to 25% of adolescents with NSP experience some degree of disability [3] and 11% may require prescription drugs to manage pain [4]. Some risk factors for adolescent NSP have been identified, including high levels of computer use [5], employment [6], negative psychosocial factors [6-8], female gender [8], and sustained postures [1]. Very low and high levels of physical activity [7] are also associated with adolescent NSP. Activity levels may influence NSP directly, or via other factors such as physical characteristics.

Physical characteristics such as muscle strength, flexibility, endurance or motor competence may be associated with spinal posture [1,7,9] or spinal stability [10], both of which may have an association with spinal pain [1,4,11]. There is some evidence that physical characteristics and adult NSP are related. Adult studies have reported that decreases in neck flexibility [12], neck endurance [12], neck muscle motor control [13], grip strength [14] and high body mass index (BMI) [15] are associated with NSP.

However the evidence for a link in adolescents is less clear. It has been noted [16] that low levels of flexibility in male adolescents and low levels of trunk endurance in female adolescents have been associated with a greater risk of "tension neck syndrome" 25 years later. Similarly, lower arm endurance in males during adolescence has been associated with more NSP in adulthood [14]. With respect to body composition, one study noted no associations between BMI at age 14 and NSP in early adulthood [17]. However, only one study to our knowledge has investigated the association of adolescent physical characteristics with NSP experienced *during *adolescence [6] and it reported no association between NSP and BMI. Salminen [18] investigated the relationship between flexibility and adolescent NSP, but this was in conjunction with LBP, and so a specific relationship with NSP was not defined. No adolescent studies have investigated the links between NSP and aerobic capacity or motor competence.

There is therefore a need for an initial exploratory study to examine the suspected links between adolescent NSP and certain physical characteristics. If it can be shown that any of these physical characteristics are related to adolescent NSP, this will provide the basis for further longitudinal work, which may in turn inform the development of specific strategies to prevent this common problem. The research question was whether lower and/or higher levels of fitness, motor competence and body composition were related to increased risk of NSP in adolescents. The physical variables used in this study reflect generalized motor performance and characteristics, rather than specific neck muscle performance, as the more general variables bear a greater relation to performance measures customarily used in schools and clinics.

## Methods

### Participants

Data were collected from 1608 adolescents (783 females, 825 males) of mean (SD) age 14.06 (0.20) yrs, who were participating in the Western Australian Pregnancy Cohort "Raine" Study . This project began with a cohort of women attending antenatal clinics at King Edward Memorial Hospital for Women, Perth, Australia between 1989 and 1991. The children have been followed at birth, 1, 2, 3, 5, 8, 10, and now 14 years of age. Inclusion criteria for the women were gestational age of 16–20 weeks, adequate English language to understand the implications of participation, and an intention to remain in Western Australia throughout follow-up. 2337 adolescents were eligible for the 14 year follow-up, and 1704 (72.9%) of these agreed to participate in some aspect of the follow-up. 1608 (68.8%) completed the data collection requirements for the analysis reported in this paper. There were no exclusion criteria for this part of the cohort. A comparison of the cohort with the Western Australian general population showed a higher proportion of high risk births, as would be expected for a major specialist hospital [19].

### Procedure

With the assistance of a research assistant, participants completed a laptop questionnaire at an assessment centre. The questionnaire contained 130 questions concerning a broad range of issues, many of which were not relevant to this study. Adolescents were asked about their experience of NSP, described as pain in the area of the posterior neck and upper trapezius, as diagrammatically defined by Kourinka et al. [20]. The relevant NSP questions were: Have you ever had neck/shoulder pain? ("yes" or "no"), Has your neck/shoulder been painful in the last month? ("yes" or "no"), and Did your neck/shoulder pain last for more than 3 months? ("yes" or "no"). The full questionnaire took about 1 hour to complete, and the NSP questions occurred in the first half. The life prevalence question is very similar to that used by Chiu and Leung [21], which was shown to be reliable and valid.

Information on diagnosed neck pain was obtained from a paper questionnaire given to the primary carer, which included the question, "Does your child have now, or has your child had in the past, any of the following health professional diagnosed medical conditions or health problems?". The primary carer had to indicate which medical diagnoses their child had experienced from a short list of general medical problems, which included "neck pain". This question was part of a questionnaire given to the primary carer, covering many other factors that are not relevant to this study.

A physical assessment of the child was carried out after the laptop questionnaire, and parts relevant to this study are described below. Height (m), body mass (kg), waist girth (cm) and arm girth (cm) were measured without shoes. Several physical performance tests were then carried out. Maximal aerobic capacity was estimated from heart rate recordings during sub-maximal cycle ergometry using the Physical Work Capacity 170 protocol [22]. The sustained back extension test [23] and the number of abdominal curls performed in 3 minutes [24,25] were used to measure trunk muscle performance. Limb muscle performance was evaluated by standing long jump [24,26], seated basketball throw [24,27] and grip strength [26,27]. Flexibility was tested using the shoulder stretch [28]. Motor competence was evaluated using the McCarron Assessment of Neuromuscular Development (MAND) [26]. The Neurodevelopmental Index (NDI) was derived from summing gender and age corrected scores from 10 tests of fine and gross motor skills, and then converting to a scale with 100 as the mean and a SD of 15. Four sub indices (muscle power, kinaesthetic integration, bimanual dexterity and persistent control) [26], each comprising two of the items, were calculated. Details of the 10 items used to generate the overall NDI and 4 sub indices are shown in table 1.

**Table 1:** Summary of MAND tests

Test	Measurement	Sub-indices
Rod slide	Smoothness and slowness of moving handle along a metre long rod, repeated both hands.	PC
Finger/nose finger	Accuracy and smoothness of index finger from nose to opposite hand's index finger, repeated both sides	PC
Hand strength	Hand grip strength with a hand dynamometer, repeated both sides	MP
Standing long jump	Distance and quality of two footed jump	MP
Heel toe walk	Quality of walking forwards and backwards along a 10 foot line	KI
Standing one leg	Time of balance on each leg with eyes open, then eyes closed.	KI
Beads on rod	Number of beads placed on rod held in non-dominant hand in 30 seconds, repeated with eyes open and closed	BD
Nut and bolt	Time to turn a large bolt, held in the dominant hand, fully onto a nut, repeated with a small bolt.	BD
Finger tapping	Number and quality of taps of index finger in 10 seconds, repeated both hands	-
Beads in box	Number of beads moved from one box to an adjacent box in 30 seconds, repeated both hands.	-

PC = Persistent Control, MP = Muscle Power, KI = Kinaesthetic Integration, BD = Bimanual Dexterity

PC = persistent control, MP = muscle power, KI = kinaesthetic integration, BD = bimanual dexterity

All of these physical performance tests have been previously validated in very similar forms [25-27,29-31] except the shoulder stretch, which has acceptable face validity. Reliability of the same or similar versions of the tests is also good [26,30-32], although there are no reports on the shoulder stretch or the basketball throw.

### Data analysis

Gender differences were analysed using independent t tests for each of the continuous variables, and Chi squared tests for the categorical variables. To facilitate the interpretation of non-linear relationships, continuous variables were banded into the bottom 25%, inter-quartile range and top 25%, and the proportion of subjects with neck pain in each segment were compared. Univariate logistic regression models predicting lifetime, last month, chronic (lasting more than 3 months) and diagnosed neck pain from each physical characteristic were calculated separately for males and females, with statistical significance set at p < 0.05, and the interquartile range of each continuous variable defined as the reference category. For the only binary variable (shoulder stretch), being able to perform the stretch was the reference category. Corrections for multiple univariate tests were not carried out as the multivariate results were the end point of the study.

Backwards stepwise likelihood ratio multivariate logistic regression models were used to evaluate the combined associations of performance factors for males and for females, with the probability for entry and removal of the likelihood ratio score statistic being p = 0.05 and 0.10 respectively. For each gender, 4 multivariate analyses for each of neck pain ever, last month, chronic and diagnosed were performed. Height and weight were included in an initial step, with abdominal curls, basketball throw, jump, back muscle endurance, PWC170, hand strength and shoulder stretch included in a second step, along with one of the body composition variables and either the NDI score or the 4 motor competence factor scores. The choice of body composition variables or motor competence variables was determined by the strength of univariate relationships with pain, and was determined for each of the eight multivariate analyses separately. The strength of the predictive ability of the model was estimated by Nagelkerke R^2^. All statistical analysis was performed using SPSS version 13.

### Ethics

Adolescents provided written informed assent and their parent/guardian provided written informed consent prior to participation. The study was approved by the Human Research Ethics Committees of Curtin University of Technology and Princess Margaret Hospital.

## Results

### Neck/shoulder pain

NSP ever was experienced by 46.8% of the participants, NSP in the past month by 28.7%, 'chronic' (lasting more than 3 months) NSP by 8.2% and diagnosed NSP by 7.1%. Females had a significantly higher prevalence of NSP ever, month and chronic, but not diagnosed NSP (see table 2). 64.9% of those with chronic NSP also had experienced NSP within the last month and 20.9% of those with chronic NSP also had diagnosed NSP according to parental report.

**Table 2:** Pain prevalence and physical test performance for males and females

**Pain variable**	**All Participants % (count) with history of pain**	**Male % (count) with history of pain**	**Female % (count) with history of pain**	**Gender difference**	
				**χ^2^**	**P**

NSP ever	46.8	41.9	52.0	16.3	<0.001
NSP in last month	28.7	22.9	34.7	27.1	<0.001
Chronic NSP	8.2	6.8	9.8	4.8	0.029
Diagnosed NSP	7.1	6.9	7.2	0.05	0.828

**Physical variable**	**All Participants mean (sd)**	**Males mean (sd)**	**Females mean (sd)**	**Gender difference**	

				**t**_**df(unless stated otherwise)**_	**P**

Height	1.64 (0.08)	1.66 (0.09)	1.62 (0.06)	-0.42_1598_	<0.001
Weight	57.7 (13.2)	58.6 (14.1)	56.7 (12.1)	-1.92_1599_	0.004
BMI	21.29 (4.15)	21.05 (4.14)	21.53 (4.16)	2.30_1598_	0.022
Waist girth (cm)	75.5 (10.8)	76.3 (11.4)	74.6 (10.1)	-3.15_1579_	0.002
Arm circumference (cm)	25.2 (3.3)	25.3 (3.4)	25.1 (3.3)	-1.17_1599_	0.244
PWC 170 score (W)	111.2 (29.9)	124.3 (31.7)	97.2 (19.9)	-19.60_1501_	<0.001
Back muscle endurance (seconds)	80.9 (60.4)	77.8 (60.1)	84.2 (60.5)	2.12_1574_	0.034
Abdominal muscle endurance (number of curls in 3 min)	21.4 (17.4)	25.4 (18.8)	17.2 (14.6)	-9.60_1569_	<0.001
Standing long jump distance (metres)	1.46 (0.29)	1.59 (0.28)	1.32 (0.23)	-20.90_1588_	<0.001
Basketball throw (metres)	5.3 (1.0)	5.7 (1.0)	4.8 (0.7)	-21.72_1583_	<0.001
Total Hand strength – right and left combined (kg)	51.8 (13.5)	57.0 (14.8)	46.3 (9.1)	-17.20_1597_	<0.001
Shoulder stretch (L) (%able)	88.9%	84.7%	92.8%	χ^2 ^= 26.1	^1 ^<0.001
NDI score	97.2 (17.4)	97.3 (18.1)	97.0 (16.6)	-0.31_1576_	0.741
Persistent control factor score	103.3 (25.4)	99.9 (26.4)	106.8 (23.7)	5.44_1594_	<0.001
Muscle Power factor score	95.9 (20.2)	102.4 (19.8)	89.2 (18.5)	-13.79_1583_	<0.001
Kinaesthetic Integration factor score	96.9 (15.2)	96.7 (15.7)	97.2 (14.7)	0.68_1596_	0.501
Bimanual Dexterity factor score	97.1 (19.3)	95.1 (19.3)	99.1 (19.1)	4.09_1598_	<0.001

Females had more NSP ever (P < 0.001, χ^2 ^= 16.26), more NSP in the past month (P < 0.001, χ^2 ^= 27.11) and more chronic NSP (P = 0.029, χ^2 ^= 4.75). There were significant gender differences for height and weight and all physical characteristics (P < 0.05) except arm circumference, kinaesthetic integration and the NDI score.

*P < 0.1, **P < 0.05, ***P < 0.01. OR = Odds Ratio, 95%CI = 95% confidence interval, IQR = interquartile range, NDI = neurodevelopmental index, MP = muscle power, PC = persistent control, BD = bimanual dexterity, PWC = Physical Work Capacity.

^1 ^group unable to do stretch is compared to group able to do stretch.

### Physical characteristics

Descriptive statistics for physical characteristics are given in table 2. Females obtained significantly higher mean scores for BMI, back endurance and the motor competence factors of Persistent Control and Bimanual Dexterity. A greater proportion of females could perform the shoulder stretch test. Males obtained significantly higher mean scores for waist girth, aerobic capacity, abdominal curl number, standing long jump, basketball throw, grip strength and the motor competence factor of muscle power. Males were also taller and heavier, with a lower BMI. There were no gender differences in arm circumference, NDI or Kinaesthetic integration.

### Associations between NSP and physical performance

#### Males

On univariate analysis, there was an increase in risk of NSP in the past month for male subjects with greatest height, an increase in risk of chronic NSP for male subjects with greatest basketball throw and NDI, and a decreased risk for chronic NSP for male subjects with the lowest weight and arm circumference. There were no significant effects on the risk of diagnosed NSP (Table 3).

**Table 3:** Univariate relationship between physical characteristics and neck/shoulder pain in males.

	Variable group	Physical variable	% with pain in each group			Log Regression Lowest 25% relative to IQR				Log Regression Highest 25% relative to IQR			
			low Q	IQR	high Q	p	OR	CI		p	OR	CI	
NSP ever	Anthropom	height	42%	40%	45%	0.635	1.08	0.78	1.52	0.235	1.24	0.87	1.76
		weight	42%	41%	44%	0.870	1.03	0.73	1.45	0.567	1.14	0.81	1.60
	Body comp	BMI	45%	40%	43%	0.198	1.25	0.89	1.76	0.472	1.13	0.81	1.60
		waist circ.	43%	42%	42%	0.808	1.04	0.74	1.46	0.910	1.02	0.72	1.44
		arm circ.	42%	41%	44%	0.792	1.05	0.75	1.46	0.409	1.16	0.82	1.63
	Aerobic	PWC 170	42%	42%	42%	0.893	1.02	0.72	1.45	0.972	1.01	0.71	1.43
	Muscle performance	back end.	38%	45%	41%	0.127	0.77	0.54	1.08	0.390	0.86	0.61	1.22
		curls	43%	41%	42%	0.513	1.12	0.80	1.58	0.690	1.07	0.76	1.52
		jump	44%	41%	41%	0.509	1.12	0.80	1.58	0.935	1.02	0.72	1.44
		throw	40%	43%	40%	0.497	0.89	0.63	1.25	0.388	0.86	0.61	1.21
		hand strength	39%	43%	43%	0.370	0.86	0.61	1.20	0.925	1.02	0.72	1.44
	Flexibility	sh stretch ^1^	34%	43%		0.083	0.70	0.47	1.04				
	Motor competence	NDI	45%	43%	36%	0.523	1.12	0.80	1.57	0.148	0.77	0.54	1.10
		PC	43%	42%	40%	0.759	1.05	0.75	1.48	0.661	0.93	0.65	1.31
		MP	41%	44%	37%	0.427	0.87	0.62	1.22	0.179	0.76	0.51	1.13
		KI	45%	40%	42%	0.208	1.23	0.89	1.71	0.751	1.06	0.73	1.56
		BD	44%	42%	38%	0.603	1.08	0.80	1.47	0.504	0.87	0.58	1.31

NSP month	Anthropom	height	21%	21%	30%	0.894	1.03	0.69	1.54	0.010**	1.69	1.13	2.50
		weight	19%	23%	27%	0.217	0.77	0.50	1.17	0.251	1.25	0.85	1.85
	Body comp	BMI	22%	23%	24%	0.850	0.96	0.65	1.44	0.709	0.92	0.58	1.46
		waist circ.	20%	23%	25%	0.356	0.83	0.55	1.24	0.656	1.09	0.74	1.63
		arm circ.	19%	25%	24%	0.100	0.71	0.48	1.07	0.805	0.95	0.64	1.42
	Aerobic	PWC 170	21%	23%	24%	0.747	0.93	0.61	1.42	0.751	1.07	0.71	1.61
	Muscle performance	back end.	20%	25%	21%	0.104	0.71	0.47	1.07	0.225	0.78	0.51	1.17
		curls	25%	20%	25%	0.139	1.36	0.91	0.20	0.150	1.35	0.90	2.02
		jump	23%	23%	23%	0.882	1.03	0.69	1.54	0.935	1.02	0.68	1.53
		throw	21%	24%	22%	0.335	0.82	0.54	1.23	0.602	0.90	0.60	1.34
		hand strength	18%	24%	26%	0.112	0.72	0.47	1.08	0.638	1.10	0.74	1.63
	Flexibility	sh stretch ^1^	19%	23%		0.275	0.76	0.47	1.23				
	Motor competence	NDI	25%	23%	20%	0.598	1.11	0.75	1.65	0.447	0.85	0.56	1.29
		PC	21%	25%	20%	0.268	0.80	0.53	1.19	0.152	0.74	0.49	1.12
		MP	20%	25%	20%	0.163	0.75	0.50	1.12	0.254	0.76	0.47	1.22
		KI	24%	23%	22%	0.765	1.06	0.72	1.56	0.753	0.93	0.59	1.46
		BD	24%	23%	22%	0.835	1.04	0.73	1.49	0.805	0.94	0.58	1.52

NSP chronic	Anthropom	height	6%	6%	9%	0.937	0.97	0.49	1.93	0.254	1.46	0.76	2.79
		weight	3%	8%	8%	0.050*	0.43	0.19	1.00	0.724	1.12	0.60	2.07
	Body comp	BMI	6%	7%	7%	0.740	0.89	0.45	1.76	0.799	0.92	0.47	1.80
		waist circ.	7%	7%	7%	0.958	1.02	0.52	1.99	0.798	1.09	0.56	2.13
		arm circ.	4%	9%	7%	0.028*	0.43	0.20	0.91	0.357	0.73	0.38	1.42
	Muscle performance	PWC 170	6%	8%	6%	0.369	0.72	0.35	1.47	0.361	0.72	0.35	1.46
		back end.	7%	6%	9%	0.453	1.29	0.66	2.54	0.183	1.56	0.81	2.99
		curls	7%	6%	8%	0.759	1.12	0.56	2.24	0.297	1.42	0.74	2.74
		jump	7%	7%	7%	0.893	0.96	0.49	1.88	0.952	1.02	0.52	2.01
		throw	6%	5%	10%	0.394	1.37	0.66	2.84	0.010**	2.33	1.22	4.44
		hand strength	5%	6%	10%	0.398	0.72	0.34	1.53	0.084	1.72	0.93	3.18
	Flexibility	sh stretch ^1^	3%	8%		0.054	0.31	0.09	1.02				
	Motor competence	NDI	8%	5%	10%	0.117	1.73	0.87	3.45	0.025*	2.13	1.10	4.12
		PC	6%	8%	6%	0.384	0.74	0.37	1.47	0.491	0.79	0.39	1.56
		MP	6%	6%	10%	0.877	0.95	0.48	1.89	0.144	1.66	0.84	3.29
		KI	7%	6%	8%	0.956	1.02	0.53	1.97	0.614	1.20	0.59	2.48
		BD	8%	6%	4%	0.317	1.34	0.75	2.39	0.255	0.57	0.21	1.51

NSP Diag	Anthropom	height	8%	7%	7%	0.690	1.14	0.60	2.17	0.991	1.00	0.50	2.03
		weight	4%	7%	9%	0.110	0.52	0.23	1.16	0.587	1.19	0.64	2.21
	Body comp	BMI	6%	7%	8%	0.628	0.84	0.42	1.69	0.765	1.10	0.58	2.12
		waist	7%	6%	9%	0.870	1.06	0.53	2.12	0.210	1.51	0.79	2.86
		arm	5%	7%	9%	0.433	0.75	0.37	1.53	0.440	1.29	0.68	2.43
	Muscle performance	PWC 170	9%	6%	6%	0.300	1.41	0.74	2.69	0.793	0.91	0.43	1.89
		BE	7%	7%	7%	0.985	1.01	0.52	1.97	0.920	0.97	0.49	1.92
		Curls	6%	6%	8%	0.934	0.97	0.48	1.98	0.462	1.28	0.66	2.50
		Jump	8%	6%	9%	0.338	1.39	0.71	2.73	0.087	1.77	0.92	3.40
		BT	5%	6%	9%	0.647	0.84	0.41	1.75	0.314	1.39	0.73	2.64
		HS	6%	8%	5%	0.319	0.71	0.37	1.39	0.226	0.64	0.31	1.32
	Flexibility	sh stretch ^1^	8%	7%		0.489	1.29	0.63	2.63				
	Motor competence	NDI	9%	6%	7%	0.230	1.49	0.78	2.86	0.497	1.27	0.64	2.53
		PC	5%	8%	5%	0.150	0.60	0.30	1.21	0.174	0.60	0.29	1.25
		MP	9%	7%	5%	0.444	1.27	0.69	2.35	0.446	0.71	0.29	1.73
		KI	7%	6%	9%	0.663	1.16	0.60	2.22	0.318	1.44	0.71	2.91
		BD	8%	7%	3%	0.572	1.18	0.67	2.09	0.110	0.42	0.14	1.22

OR = Odds Ratio, 95%CI = 95% confidence interval, IQR = interquartile range, BMI = body mass index, PWC = physical work capacity, SS = shoulder stretch (L), NDI = neurodevelopmental index, PC = persistent control, MP = muscle power, KI = kinesthetic integration, BD = bimanual dexterity. *P < 0.05, **P < 0.01

*P < 0.1, **P < 0.05, ***P < 0.01. OR = Odds Ratio, 95%CI = 95% confidence interval, IQR = interquartile range, NDI = neurodevelopmental index, MP = muscle power, PC = persistent control, BD = bimanual dexterity, PWC = Physical Work Capacity.

^1 ^group unable to do stretch is compared to group able to do stretch.

For multivariate analysis, the common variables described in the methods section were entered, together with BMI and NDI for the NSP ever analysis, arm circumference and the four MAND factors for the NSP month analysis, arm circumference and NDI for the NSP chronic analysis, and waist circumference and the four MAND factors for the NSP diagnosed analysis, according to the strength of univariate relationships. Males in the lowest quartile of back muscle endurance were less likely to have NSP ever, and there was a similar trend for those in the highest quartile of NDI. There were no multivariate associations between physical characteristics and male NSP in the past month. Males in the highest quartile of basketball throw distance were more likely to have chronic NSP and there was a trend for those unable to do the shoulder stretch to be less likely to have chronic NSP. Males in the highest quartile of jump distance were more likely to have diagnosed NSP, but those in the highest quartile of muscle power were less likely to have diagnosed NSP. Those in the lowest quartile of persistent control were less likely to have NSP. Nagelkerke R^2 ^of logistic regression models ranged from 0.019 to 0.085 (Table 4).

**Table 4:** Multivariate relationships between physical characteristics and each type of neck/shoulder pain in males and females.

**Gender**	**NSP variable**	**Physical characteristics associating with NSP (at P < 0.1)**	**Lowest 25% relative to IQROR, (95% CI)**	**Highest 25% relative to IQR,OR (95% CI)**
Male	NSP ever	NDI	1.26 (0.87–1.83)	0.73 (0.50–1.1)*
		back endurance	0.66 (0.46–0.97)**	0.82 (0.57–1.18)
	NSP month	-	-	-
	NSP chronic	throw	1.96 (0.87–4.45)	2.47 (1.22–5.00)**
		shoulder stretch	^1^0.30 (0.09–1.01)*	NA
	NSP diagnosed	jump	0.75 ((0.27–2.07)	3.47 (1.55–7.74)***
		MP	1.92 (0.70–5.30)	0.33 (0.12–0.94)**
		PC	0.42 (0.19–0.95)**	0.69 (0.33–1.46)

Female	NSP ever	Abdominal curls	1.36 (0.94–1.97)	1.57 (1.07–2.311)**
		throw	0.97 (0.66–1.42)	0.60 (0.40–0.90)**
		shoulder stretch	^1^0.54 (0.30–0.98)**	NA
	NSP month	throw	1.27 (0.84–1.90)	0.53 (0.34–0.84)***
		jump	0.61 (0.41–0.92)**	0.70 (0.46–1.06)*
		BD	0.86 (0.58–1.26)	1.77 (1.18–2.65)***
		PWC	0.751 (0.50–1.13)	0.61 (0.40–0.93)**
	NSP chronic	-	-	-
	NSP diagnosed	back endurance	2.12 (1.20–3.74)**	2.12 (1.18–3.83)**

*P < 0.1, **P < 0.05, ***P < 0.01. OR = Odds Ratio, 95%CI = 95% confidence interval, IQR = interquartile range, NDI = neurodevelopmental index, MP = muscle power, PC = persistent control, BD = bimanual dexterity, PWC = Physical Work Capacity.

^1 ^group unable to do stretch is compared to group able to do stretch.

#### Females

On univariate analysis there was an increase in risk for NSP in the past month for females with the highest bimanual dexterity, and an increase in risk of chronic NSP for females with the lowest hand strength. There was a decreased risk of NSP ever for females with the highest basketball throw, and a decreased risk of NSP in the past month for females with highest PWC170, and lowest and highest jump distance. There were no significant effects on the risk of diagnosed NSP (Table 5).

**Table 5:** Univariate relationship between physical characteristics and neck/shoulder pain in females.

	Variable group	Physical variable	% with pain in each group			Log Regression Lowest 25% relative to IQR				Log Regression Highest 25% relative to IQR			
			low Q	IQR	high Q	p	OR	CI		p	OR	CI	
NSP ever	Anthropom	height	50%	52%	52%	0.658	0.93	0.66	1.3	0.955	0.99	0.7	1.41
		weight	51%	53%	51%	0.681	0.93	0.66	1.31	0.564	0.9	0.64	1.28
	Body comp	BMI	54%	52%	49%	0.617	1.09	0.77	1.54	0.501	0.89	0.63	1.26
		waist circ.	51%	52%	52%	0.779	0.95	0.68	1.34	0.979	1	0.7	1.41
		arm circ.	52%	53%	48%	0.800	0.96	0.69	1.33	0.302	0.82	0.57	1.19
	Aerobic	PWC 170	50%	55%	48%	0.314	0.83	0.58	1.19	0.136	0.76	0.53	1.09
	Muscle performance	back end.	51%	51%	55%	0.878	0.97	0.69	1.38	0.389	1.17	0.82	1.65
		curls	54%	48%	56%	0.181	1.26	0.9	1.78	0.091	1.36	0.95	1.94
		jump	47%	55%	51%	0.069	0.73	0.52	1.03	0.351	0.85	0.6	1.2
		throw	54%	54%	44%	0.988	1	0.71	1.41	0.039*	0.68	0.47	0.98
		hand strength	51%	51%	55%	0.975	1.01	0.72	1.41	0.312	1.2	0.84	1.71
	Flexibility	sh stretch ^1^	40%	53%		0.074	0.60	0.34	1.05				
	Motor competence	NDI	48%	54%	53%	0.137	0.77	0.55	1.09	0.848	0.97	0.68	1.37
		PC	47%	55%	52%	0.079	0.75	0.54	1.03	0.617	0.9	0.6	1.35
		MP	49%	56%	48%	0.118	0.77	0.55	1.07	0.080	0.73	0.51	1.04
		KI	51%	52%	55%	0.840	0.97	0.69	1.35	0.583	1.12	0.75	1.65
		BD	50%	51%	57%	0.975	1	0.72	1.38	0.163	1.29	0.9	1.85

NSP month	Anthropom	height	35%	35%	34%	0.951	0.99	0.69	1.41	0.723	0.94	0.65	1.35
		weight	36%	34%	34%	0.701	1.07	0.75	1.53	0.923	0.98	0.68	1.41
	Body comp	BMI	37%	35%	31%	0.567	1.11	0.78	1.59	0.431	0.86	0.6	1.25
		waist circ.	37%	34%	32%	0.419	1.16	0.81	1.66	0.723	0.94	0.65	1.36
		Arm circ.	38%	25%	31%	0.476	1.13	0.81	1.59	0.347	0.83	0.56	1.23
	Aerobic	PWC 170	34%	38%	29%	0.331	0.83	0.57	1.21	0.031*	0.65	0.44	0.96
	Muscle performance	back end.	34%	35%	34%	0.798	0.95	0.66	1.37	0.711	0.93	0.65	1.35
		curls	33%	34%	37%	0.833	0.96	0.67	1.38	0.464	1.15	0.79	1.66
		jump	31%	40%	30%	0.033*	0.68	0.47	0.97	0.024*	0.65	0.45	0.95
		throw	38%	36%	28%	0.605	1.1	0.77	1.56	0.052	0.67	0.45	1
		hand strength	33%	36%	35%	0.445	0.87	0.61	1.24	0.803	0.95	0.66	1.38
	Flexibility	Sh stretch ^1^	31%	35%		0.574	0.85	0.47	1.66				
	Motor competence	NDI	30%	35%	39%	0.285	0.82	0.57	1.18	0.272	1.22	0.85	1.76
		PC	30%	36%	38%	0.075	0.73	0.52	1.03	0.725	1.08	0.71	1.63
		MP	35%	36%	31%	0.689	0.93	0.66	1.32	0.240	0.8	0.54	1.17
		KI	31%	36%	38%	0.275	0.82	0.57	1.17	0.655	1.1	0.73	1.64
		BD	31%	33%	44%	0.594	0.91	0.64	1.29	0.008**	1.64	1.14	2.37

NSP chronic	Anthropom	height	11%	9%	9%	0.517	1.2	0.69	2.1	0.941	0.98	0.53	1.8
		weight	12%	9%	9%	0.183	1.46	0.84	2.53	0.772	1.09	0.6	1.99
	Body comp	BMI	12%	8%	10%	0.091	1.63	0.93	2.86	0.323	1.35	0.75	2.43
		waist circ.	11%	9%	11%	0.350	1.32	0.74	2.33	0.282	1.37	0.77	2.44
		arm circ.	12%	9%	10%	0.237	1.38	0.81	2.37	0.553	1.21	0.65	2.26
	Muscle performance	PWC 170	9%	10%	11%	0.576	0.84	0.45	1.55	0.929	1.03	0.57	1.84
		back end.	10%	9%	10%	0.604	1.17	0.65	2.09	0.800	1.08	0.59	1.97
		curls	12%	8%	9%	0.177	1.48	0.84	2.61	0.785	1.09	0.58	2.05
		jump	9%	11%	9%	0.410	0.78	0.44	1.4	0.415	0.78	0.42	1.43
		throw	10%	10%	10%	0.943	1.02	0.57	1.82	0.842	1.06	0.58	1.97
		hand strength	13%	7%	11%	0.032*	1.85	1.05	3.25	0.114	1.62	0.89	2.95
	Flexibility	sh stretch ^1^	7%	10%		0.572	0.74	0.26	2.13				
	Motor competence	NDI	11%	10%	8%	0.617	1.15	0.66	2.02	0.654	0.87	0.47	1.61
		PC	8%	10%	11%	0.362	0.77	0.43	1.36	0.665	1.15	0.61	2.18
		MP	11%	10%	8%	0.770	1.09	0.63	1.88	0.477	0.79	0.42	1.51
		KI	9%	11%	7%	0.389	0.78	0.43	1.38	0.186	0.61	0.29	1.27
		BD	11%	9%	9%	0.398	1.27	0.73	2.18	0.840	1.07	0.58	1.98

NSP diagnosed	Anthropom	height	6%	8%	8%	0.429	0.76	0.38	1.52	0.994	1.00	0.51	1.94
		weight	7%	7%	7%	0.800	0.92	0.46	1.81	0.980	0.99	0.51	1.93
	Body comp	BMI	8%	7%	7%	0.551	1.22	0.63	2.38	0.725	1.13	0.57	2.23
		waist circ.	8%	7%	7%	0.854	1.07	0.55	2.07	0.785	0.91	0.45	1.83
		arm circ.	8%	7%	7%	0.516	1.23	0.66	2.30	0.901	1.05	0.50	2.18
	Muscle performance	PWC 170	5%	8%	7%	0.226	0.62	0.29	1.34	0.791	0.91	0.46	1.81
		back end.	9%	6%	8%	0.109	1.71	0.89	3.29	0.275	1.47	0.74	2.92
		curls	5%	6%	10%	0.520	0.78	0.37	1.67	0.167	1.58	0.83	3.03
		jump	5%	8%	7%	0.196	0.63	0.31	1.27	0.433	0.76	0.38	1.51
		throw	7%	7%	8%	0.695	1.14	0.58	2.25	0.460	1.30	0.65	2.60
		hand strength	6%	7%	8%	0.467	0.77	0.38	1.56	0.754	1.11	0.58	2.15
	Flexibility	sh stretch ^1^	11%	7%		0.220	1.78	0.71	4.32				
	Motor competence	NDI	7%	8%	6%	0.547	0.81	0.41	1.60	0.400	0.74	0.36	1.51
		PC	6%	7%	10%	0.555	0.82	0.42	1.60	0.272	1.49	0.73	3.01
		MP	6%	7%	8%	0.675	0.86	0.43	1.72	0.656	1.17	0.59	2.29
		KI	6%	8%	6%	0.441	0.77	0.39	1.51	0.510	0.77	0.35	1.70
		BD	8%	7%	7%	0.536	1.22	0.65	2.32	0.695	1.15	0.57	2.33

OR = Odds Ratio, 95%CI = 95% confidence interval, IQR = interquartile range, BMI = body mass index, PWC = physical work capacity, SS = shoulder stretch (L), NDI = neurodevelopmental index, PC = persistent control, MP = muscle power, KI = kinesthetic integration, BD = bimanual dexterity.

*P < 0.05, **P < 0.01 *P < 0.1, **P < 0.05, ***P < 0.01. OR = Odds Ratio, 95%CI = 95% confidence interval, IQR = interquartile range, NDI = neurodevelopmental index, MP = muscle power, PC = persistent control, BD = bimanual dexterity, PWC = Physical Work Capacity.

^1 ^group unable to do stretch is compared to group able to do stretch.

For multivariate analysis, the common variables described in the methods section were entered, together with arm circumference and the four MAND factors for the NSP ever analysis and the NSP month analysis, and BMI and the four MAND factors for the NSP chronic and diagnosed analysis, according to the strength of univariate relationships. Females in the highest quartile of basketball throw were less likely to have NSP ever, and females in the highest quartile of abdominal curls were more likely to have NSP. Females unable to do the shoulder stretch were less likely to have NSP. Females in the lowest quartile of jump distance were less likely to have NSP in the past month. Females in the highest quartile of basketball throw, bimanual dexterity and PWC170 were less likely to have NSP in the past month. There was also a trend for those in the highest quartile of jump distance to be less likely to have NSP in the past month. There were no multivariate associations between physical characteristics and chronic NSP in females. Females in the lowest quartile of back endurance were more likely to have diagnosed NSP, and those in the highest quartile of back endurance were more likely to have diagnosed NSP. The Nagelkerke R^2 ^of logistic regression models ranged from 0.001 to 0.064 (Table 4)

## Discussion

NSP is clearly a common problem in adolescents, with this study showing a prevalence of pain similar to that reported in other adolescent studies [1,2]. That almost one in ten adolescents have experienced NSP of at least 3 months duration is a strong indicator that adolescent NSP is a significant problem. The search for adolescent risk factors is therefore of great importance, so that effective prevention and management can be implemented. This study is the first to suggest that some physical characteristics are associated with adolescent NSP, although the strength of these associations was weaker than anticipated.

### Cross-sectional data

This study analysed cross-sectional data only, so relationships identified could be the result of causality in both directions: NSP could be influenced by physical characteristics or vice versa. There is evidence that adults with back pain may experience a 'deconditioning' effect associated with pain inhibiting and restricting participation in work, leisure and household activities [33]. In contrast, there is evidence that poor back muscle endurance increases the risk of back pain episodes in manual workers [34].

Longitudinal data (currently being collected on this cohort) is required to elucidate the direction of any relationship. The remainder of this section discusses the cross-sectional results and suggests potential mechanisms for observed relationships.

### Body composition

Although a weak univariate relationship between low arm circumference and a lower risk of chronic NSP was observed in males, body composition was not associated with any form of NSP in either gender after multivariate analysis. This concurs with previous adolescent findings [35] and underlines the importance of multivariate analysis with a comprehensive range of covariates.

### Aerobic capacity

Higher aerobic capacity, after correction for other variables including body weight, was associated with a lower risk of NSP in the last month for females only, with a similar trend in NSP ever. The lower risk of NSP with improved aerobic capacity for NSP in females may be associated with increased levels of physical activity which is known to sometimes have a beneficial effect on spinal pain disorders [7]. This may also relate to a deconditioning mechanism, where females with NSP reduce their participation in physical activity and lose aerobic capacity. The lack of any relationships for males may indicate a differing mechanism or response to neck pain based on gender.

### Muscle performance

There were inconsistent associations between arm muscle performance and NSP after multivariate analysis. Greater upper body power, as measured by the basketball throw, was protective in females for both NSP ever and in the past month, but a risk factor for chronic NSP in males. The reason for this gender difference is unclear although other factors such as specific sport participation may influence these findings. Females (but not males) engaging in more dynamic arm activities have less pain [8,36], and given that greater amounts of dynamic arm activities may increase upper body power, this may explain the pattern in females. The opposite pattern in males, with increased risk of chronic NSP in the most powerful quartile, may partly relate to greater arm activity not having a protective effect in males [8,33], and also because their high arm power may be a proxy for greater overall physical activity levels (not just upper limb activity), which relates to greater NSP in males [37]. In contrast, Barnekow-Bergvist et al. [14] reported that greater arm endurance in adolescent males was related to a reduced risk of NSP in adulthood, which may relate to a deconditioning effect secondary to NSP.

Multivariate associations between NSP and leg power were very different to those with arm power. In females, a low jump performance decreased risk of NSP in the past month, effectively the opposite effect seen with upper body power. Aurvinen et al. [38] reported that higher overall activity levels may increase NSP risk in females. Since it is possible that higher overall activity may be associated with greater leg power, this may explain our finding of low leg power reducing risk. Although differing effects on NSP from arm activity levels and overall activity levels may initially appear paradoxical, it is possible that the relationship between overall activity levels and NSP is not direct but mediated by performance in sports that may increase risk of NSP. Similarly, diagnosed neck pain was associated with greater jump distance in males, although this was not observed for the other pain variables. This result may indicate a similar mechanism to that described in females.

A very similar pattern was observed between abdominal endurance and NSP ever after multivariate analysis, with better performance associated with greater risk of pain in females only. Mechanisms may be similar to those described for leg power. In contrast, Mikkelson et al. [16] reported that poorer female adolescent abdominal endurance was a risk factor. However, Mikkelson et al. [16] reported these outcomes in adulthood.

Less back muscle endurance was associated with a decreased risk of NSP ever in males after multivariate analysis, which was analogous to the findings for leg power and abdominal endurance in females, and may again relate to the males being involved in more vigorous physical activity [37]. Similarly, females with a diagnosis of NSP were more likely to have high back endurance, and this could relate to greater overall activity levels, as previously described. However, females with low back endurance also had a higher risk of diagnosed NSP. It is possible that these females were below a threshold of endurance at which any effects on spinal stability became important, or alternatively were experiencing a deconditioning effect as a result of the pain. However, this effect was not seen in males, who had lower back endurance overall.

### Flexibility

One surprising multivariate finding was that poorer shoulder girdle flexibility, as measured by the shoulder stretch, was related to a significantly decreased risk of NSP in the past month in females. There was also a strong trend for the same effect on chronic NSP in males. Though counter-intuitive, there are reports of a relationship between lower shoulder rotational flexibility and greater upper limb activity levels in elite adult water polo [39] and volleyball players [40]. Greater amounts of dynamic upper limb activity have also been shown to reduce the risk of female adolescent NSP [8,36] and so these separate findings may explain the overall association of reduced flexibility and lower risk of NSP observed in this study.

### Motor competence

Males with higher levels of the motor competence factor of muscle power had a reduced risk of diagnosed NSP, and there was a trend for higher overall motor competence (NDI) to be associated with lower risk of NSP ever in males after multivariate analysis. This was expected, given that higher motor competence might have a protective effective on the musculoskeletal system [41]. However this relationship may be weakened by males with better motor competence being more involved in vigorous activities, as suggested by evidence that pre-pubescent children with higher motor competence engage in more vigorous play [42], and thus more likely to develop NSP [37]. This potential confounding may possibly explain the contradictory finding of lower persistent control being associated with a lower risk of diagnosed NSP. In contrast, poorer coordination may be a result of reduced motor practice as part of a reduction of activities associated with NSP.

In females, higher bimanual dexterity significantly increased risk of NSP in the past month. Bimanual dexterity relates to the co-ordination of fine motor skills across both arms, and might be developed by activities such as playing musical instruments, needlework, computer work or craftwork, which are known risk factors for female adolescent NSP [36].

### Strength of associations

Evidence from longitudinal studies [14,16] demonstrates that physical performance in adolescence can influence the development of NSP in adulthood, although a deconditioning response to the presence of NSP is also possible. The predictive utility of the models in the current study was very low however, with Nagelkerke R^2 ^ranging from only 0.001 to 0.085. The lack of stronger relationships was not due to missed curvilinear relationships as these were accounted for in the analysis, and the study was not underpowered as weak relationships were detected.

This may indicate that either physical performance is not strongly related to NSP during adolescence or that the direction and mechanisms are more complex and other factors need to be considered. One of the strengths of our study was the broad range of physical variables adjusted for in the analyses, but certain possible confounders such as activity levels and sport participation were not included in the current analyses. Consideration of these may have either reduced or strengthened observed relationships, and should be attempted in further work. In addition, NSP was treated as a homogenous entity, but in reality it may have several sub-groups with different aetiologies. Real, but differing, associations between physical performance and each sub-group may thus have been lost in the current analysis. Further work towards subgroup identification is intended.

NSP is not a simple construct and thus four measures were used, including a parental report of health professional diagnosed NSP. Whilst parental report of diagnosed neck pain has limited detail and accuracy, it reinforced the self-report measure of NSP. Further, strong relationships could be expected to be more consistent across the different measures.

Associations were very different across genders, with no common effects seen. These differing gender effects may be the result of differences in the type and vigour of sporting activities [43], as well as anthropometric differences, and possible variation in underlying pain mechanisms and psychosocial effects. Whatever the cause, these differences emphasise the need to continue to consider gender in future work, as gender will be a possible confounder of many pain/physical characteristics relationships.

## Conclusion

NSP is clearly an important health issue for adolescents. Some aspects of physical performance are associated with adolescent NSP. Interestingly, better performance sometimes increased rather than decreased risk, suggesting that the direction and mechanisms are complex. Associations differed between genders, suggesting that NSP in males and females may have different mechanisms, or that these factors may interact differently. However, despite the large sample and examination of curvilinear relationships, multiple physical factors and gender specific effects, the associations were weak suggesting complex mechanisms for NSP development.

## Competing interests

The authors declare that they have no competing interests.

## Authors' contributions

MCP analysed data, drafted the manuscript and assisted with final approval. LMS designed the study, designed and revised the manuscript and assisted with final approval. PBO designed the study, revised the manuscript and assisted with final approval. AJS analysed data, revised the manuscript and assisted with final approval. BH designed the study, revised the manuscript and assisted with final approval.

## Pre-publication history

The pre-publication history for this paper can be accessed here:


